# Predictors of Acute Postsurgical Pain following Gastrointestinal Surgery: A Prospective Cohort Study

**DOI:** 10.1155/2021/6668152

**Published:** 2021-01-28

**Authors:** Qing-Ren Liu, Mu-Huo Ji, Yu-Chen Dai, Xing-Bing Sun, Cheng-Mao Zhou, Xiao-Dong Qiu, Jian-Jun Yang

**Affiliations:** ^1^School of Medicine, Southeast University, Nanjing 210009, China; ^2^Department of Anesthesiology, Xishan People's Hospital of Wuxi City, Wuxi 214105, China; ^3^Department of Anesthesiology, The Second Affiliated Hospital, Nanjing Medical University, Nanjing 210011, China; ^4^Department of Anesthesiology, Zhongda Hospital, Medical School, Southeast University, Nanjing 210009, China; ^5^Department of Anesthesiology,Pain and Perioperative Medicine, The First Affiliated Hospital of Zhengzhou University, Zhengzhou 450000, China

## Abstract

**Background:**

Several predictors have been shown to be independently associated with chronic postsurgical pain for gastrointestinal surgery, but few studies have investigated the factors associated with acute postsurgical pain (APSP). The aim of this study was to identify the predictors of APSP intensity and severity through investigating demographic, psychological, and clinical variables.

**Methods:**

We performed a prospective cohort study of 282 patients undergoing gastrointestinal surgery to analyze the predictors of APSP. Psychological questionnaires were assessed 1 day before surgery. Meanwhile, demographic characteristics and perioperative data were collected. The primary outcomes are APSP intensity assessed by numeric rating scale (NRS) and APSP severity defined as a clinically meaningful pain when NRS ≥4. The predictors for APSP intensity and severity were determined using multiple linear regression and multivariate logistic regression, respectively.

**Results:**

112 patients (39.7%) reported a clinically meaningful pain during the first 24 hours postoperatively. Oral morphine milligram equivalent (MME) consumption (*β* 0.05, 95% CI 0.03–0.07, *p* < 0.001), preoperative anxiety (*β* 0.12, 95% CI 0.08–0.15, *p* < 0.001), and expected postsurgical pain intensity (*β* 0.12, 95% CI 0.06–0.18, *p* < 0.001) were positively associated with APSP intensity. Furthermore, MME consumption (OR 1.15, 95% CI 1.10–1.21, *p* < 0.001), preoperative anxiety (OR 1.33, 95% CI 1.21–1.46, *p* < 0.001), and expected postsurgical pain intensity (OR 1.36, 95% CI 1.17–1.57, *p* < 0.001) were independently associated with APSP severity.

**Conclusion:**

These results suggested that the predictors for APSP intensity following gastrointestinal surgery included analgesic consumption, preoperative anxiety, and expected postsurgical pain, which were also the risk factors for APSP severity.

## 1. Introduction

Acute postsurgical pain (APSP) is defined as pain within the first 24 hours postoperatively. Moderate or severe APSP is reported in 10% to 57% of patients undergoing various types of surgeries [1]. A larger study with 22,000 patients also showed that 28% experienced moderate–severe APSP after various types of surgeries [2]. APSP has a considerable influence on cardiovascular system, respiratory system, and immune system, which impacts postoperative early recovery [3]. Furthermore, APSP can transit to CPSP that may impair a patient's long-term quality of life [4, 5]. It has been demonstrated that female [6], young age [2, 7, 8], preoperative anxiety [7, 9–15] and depression [10], expected postsurgical pain [9, 16–19], pain catastrophizing [7, 20, 21], preoperative pain [7, 10, 22], and type of surgery [14, 21, 23] are risk factors for APSP.

Enhanced Recovery after Surgery (ERAS) is a multimodal perioperative care pathway designed to reduce perioperative stress, maintain postoperative physiological function, and accelerate recovery after surgery. Thus, major complications can be reduced while length of stay can be shortened after major gastrointestinal surgery [24–28]. Pain management is an integral part of ERAS protocols. Around 30% of patients reported that pain would affect mood, sleep, and enjoyment of life after gastrointestinal surgery while 12% visited the emergency department because of unendurable pain [29]. Therefore, great attention should be paid to early intervention and control of acute pain through identifying specific predictors after gastrointestinal surgery [30–32]. Currently, several studies on predictors of CPSP have been performed among patients undergoing gastrointestinal surgery, showing that the associated predictors are female gender [30], younger age [30], preoperative anxiety and depression [29], and preoperative pain [33]. However, little is known about the predictors of APSP intensity or severity for gastrointestinal surgery.

The purpose of our study was to determine the predictors of APSP intensity and severity for gastrointestinal surgery by investigating demographic, psychological, and clinical variables. The primary outcome of interest is APSP intensity assessed using the 11-point numeric rating scale (NRS, 0 = no pain at all, 10 = strongest pain imaginable) [34, 35] and APSP severity defined as a clinically meaningful pain when maximum pain NRS ≥4 [12, 14]. We hypothesized that analgesic consumption and certain psychological variables would be the independent risk factors for APSP after controlling the confounding factors.

## 2. Methods

### 2.1. Participants

The protocol was registered at the Chinese Clinical Trial Registry (ChiCTR1900024837). This prospective cohort study was carried out at Zhongda Hospital, Southeast University. The study was approved by IEC for Clinical Research of Zhongda Hospital Affiliated to Southeast University (No. 2019ZDSYLL084-P01). Patients eligible for participation were those scheduled for gastrointestinal surgery between August 2019 and January 2020. The study recruited 345 patients to investigate the prediction of acute pain after gastrointestinal surgery. The written informed consent was obtained as a condition to participate in the study. Inclusion criteria included patients undergoing elective gastrointestinal surgery, aged ≥18 years old and physical status classification of the American Society of Anesthesiologists (ASA) ≤III. Patients with self-expression disorder and hearing or visual impairment were excluded.

### 2.2. Demographic Characteristics

Demographic characteristics such as gender, age, height, weight, calculated body mass index (BMI), occupation status, education level, smoking (never, former, and current), alcohol drinking (never, former, and current), hypertension, diabetes, coronary heart disease, and physical status classification of ASA were collected on the day before surgery. Moreover, patients were asked to rate a history of preexisting pain. Patients who suffered from pain for more than 3 months were classified as having previous chronic pain. Simultaneously, a history of previous surgery was also attained and participants were classified as previous surgery and no previous surgery.

### 2.3. Psychological Questionnaires

During the preoperative visit, psychological factors were assessed with the respective standardized and validated questionnaires: Hospital Anxiety and Depression Scale (HADS) [36] and Surgical Fear Questionnaire (SFQ) [37]. The HADS consists of two seven-item subscales that measure anxiety and depression levels among patients in nonpsychiatric hospital settings. Subscale scores vary between 0 and 21, with higher scores representing higher levels of anxiety and depression. The SFQ, as a valid and reliable index of surgical fear, consists of two eight-item subscales that measure short-term consequences of surgery (SFQ-s) and fear of the long-term consequences (SFQ-l). All item scores covered the full range of 0–10. In addition, patients were asked to rate their average expected pain intensity for the first day postoperatively (on a scale from 0 = no pain at all to 10 = strongest pain imaginable) [9].

### 2.4. Surgery and Anesthesia

Surgery was performed by 3 surgeons according to a standardized protocol. Data regarding type, site, duration of surgery, as well as number of drainage tube and length of hospital stay, were collected. Additionally, preoperative clinical factors were attained from the medical record. The primary sites of surgery included stomach, colorectum, and small bowel. The surgery was performed using either open or laparoscopic procedure.

After standard monitoring (electrocardiogram, peripheral oxygen saturation, noninvasive blood pressure, and end-tidal carbon dioxide), a standardized anesthetic protocol was carried out by the anesthesiologists who were members of the research team. General anesthesia was induced with intravenous (IV) administration of 0.3 *μ*g/kg of sufentanil, 2 to 2.5 mg/kg of propofol, and 0.6 mg/kg of rocuronium. Anesthesia was maintained with sevoflurane, remifentanil, and cisatracurium. During skin closure, sufentanil (0.1 *μ*g/kg, IV) was given.

### 2.5. Acute Postoperative Pain

The primary outcome was the maximum pain intensity assessed using NRS pain scores within the first 24 h after surgery. At 6–8, 14–16, and 22–24 hours after operation, patients were asked to report their level of pain during movement (changing position to either sitting up or standing), respectively. The maximum pain intensity was identified as the highest NRS pain score at three time points within the first 24 h postoperatively. We defined NRS ≥4 of the maximum pain as clinically meaningful pain, indicating moderate-to-severe pain intensity.

After surgery, patients received routine postoperative pain management. In the postanesthesia care unit (PACU), patients received tramadol (50 mg, IV) or oxycodone (2 mg, IV) when NRS pain at rest ˃ 3. On the ward, patients received celecoxib 200 mg orally every 12 h, as well as a patient-controlled analgesia device with oxycodone or sufentanil. Furthermore, patients administered the breakthrough pain using tramadol intravenously only as needed. The analgesic consumption of 24 hours postoperatively was measured from arriving PACU. To compare the analgesic consumption, systemic opioid analgesics were converted to oral milligram morphine equivalent (MME). MME use was calculated using standard published conversion factors.

### 2.6. Statistical Analysis

First, descriptive statistics were calculated on all variables. Mean ± standard deviation was used for normally distributed variables, medians (interquartile range) for nonnormally distributed variables, and number (percentage) for categorical data. *p* < 0.05 was considered statistically significant. Pearson's test was performed to investigate the correlation between preoperative anxiety and depression, expected postsurgical pain, SFQ-s, SFQ-l, and APSP intensity. Next, univariate linear regression and multiple linear regression models were used to examine whether MME consumption, preoperative anxiety, and expected postsurgical pain had an independent effect on APSP intensity. Furthermore, we also performed univariate logistic regression and multivariate logistic regression to identify whether MME consumption, preoperative anxiety, and expected postsurgical pain were independently associated with APSP severity. All analyses were performed with *R* (http://www.R-project.org) and EmpowerStats software (http://www.empowerstats.com, X&Y Solutions, Inc., Boston, MA, USA).

## 3. Results

**Figure 1:**
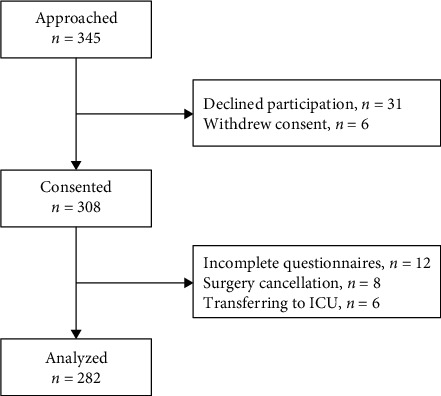
Flowchart of the study. ICU: intensive care unit.

345 patients were approached for participation in this study. 31 declined participation, and 6 withdrew consent. There were 12 patients excluded owing to incomplete questionnaires, 8 patients for surgery cancellation, and 6 patients for transferring to intensive care unit. Thus, 282 patients (81.7%) were available for analysis (Figure 1).

### 3.1. Demographic, Clinical, and Psychological Characteristics

Demographic and clinical characteristics of patients are shown in Table 1. 110 patients (39%) were female; 38 (13.5%) were single, separated or divorced; 183 (64.9%) had a middle-school education or above; 66 (23.4%) reported previous chronic pain; and 143 (50.7%) had a history of previous surgery. Most patients (93.2%) received gastric or colorectal surgery, but only 83 patients (29.4%) underwent laparoscopic surgery. Most patients (84.4%) had malignant tumor.

Psychological characteristics of patients are shown in Table 2. In preoperative assessment, 56 patients (19.9%) had anxiety, and 39 patients (13.8%) had depression. The median score of expected postsurgical pain intensity was 5.0 (3.0, 7.0), and 113 patients (40.1%) reported moderate-to-severe pain (NRS ≥4) during movement within the first 24 hours postoperatively. The median scores of SFQ-s and SFQ-l subscales were 9.0 (3.0, 15.0) and 6.0 (2.0, 14.0), respectively.

### 3.2. Predicting Postsurgical Pain Intensity

Table 3 presented Pearson's correlation coefficients between APSP intensity and other study variables. APSP intensity was significantly correlated with MME consumption (Pearson's *r* = 0.63, *p* < 0.001), preoperative anxiety (Pearson's *r* = 0.48, *p* < 0.001), preoperative depression (Pearson's *r* = 0.36, *p* < 0.001), and expected postsurgical pain intensity (Pearson's *r* = 0.38, *p* < 0.001). Meanwhile, it was also obviously correlated with SFQ-s (Pearson's *r* = 0.31, *p* < 0.001) and SFQ-l (Pearson's *r* = 0.32, *p* < 0.001) but negatively correlated with age (Pearson's *r* = −0.17, *p* < 0.001).

As shown in Table 4, for the univariate analysis, APSP intensity was significantly correlated with MME consumption (*β* 0.07, 95% CI 0.05–0.09, *p* < 0.001), preoperative anxiety (*β* 0.15, 95% CI 0.12–0.18, *p* < 0.001), preoperative depression (*β* 0.12, 95% CI 0.08–0.15, *p* < 0.001), and expected postsurgical pain (*β* 0.20, 95% CI 0.15–0.26, *p* < 0.001). In addition, SFQ-s (*β* 0.04, 95% CI 0.03–0.05, *p* < 0.001) and SFQ-l (*β* 0.04, 95% CI 0.03–0.06, *p* < 0.001) were associated with APSP intensity. Meanwhile, age (*β* −0.02, 95% CI: 0.03 to −0.01, *p*=0.004) and occupation (*β* 0.38, 95% CI 0.07–0.70, *p*=0.018) might also be associated with APSP intensity.

To determine the predictors of postsurgical pain intensity, a multiple linear regression analysis was conducted (Table 4). Preoperative anxiety (*β* 0.12, 95% CI 0.08–0.15, *p* < 0.001), expected postsurgical pain (*β* 0.12, 95% CI 0.06–0.18, *p* < 0.001), and MME consumption were positively associated with APSP intensity.

### 3.3. Risk Factors for Postsurgical Pain Severity

169 patients reported absence of pain or mild pain (NRS ≤3) on APSP severity, whereas 113 patients reported moderate-to-severe pain, regarded as clinically meaningful pain. The univariate logistic analysis showed that APSP severity was significantly correlated with MME consumption (OR 1.18, 95% CI 1.12–1.24, *p* < 0.001), preoperative anxiety (OR 1.40, 95% CI 1.28–1.53, *p* < 0.001), preoperative depression (OR 1.31, 95% CI 1.20–1.43, *p* < 0.001), and expected postsurgical pain (OR 1.55, 95% CI 1.36–1.78, *p* < 0.001). In addition, SFQ-s (OR 1.09, 95% CI 1.06–1.12, *p* < 0.001), SFQ-l (OR 1.09, 95% CI 1.05–1.12, *p* < 0.001), and age (OR 0.97, 95% CI 0.95–0.99, *p*=0.005) were also associated with APSP severity (Table 5).

After multivariable risk adjustment for potential confounding factors, a logistic regression was performed (Table 5). MME consumption (OR 1.15, 95% CI 1.10–1.21, *p* < 0.001), preoperative anxiety (OR 1.33, 95% CI 1.21–1.46, *p* < 0.001), and expected postsurgical pain (OR 1.36, 95% CI 1.17–1.57, *p* < 0.001) were independently associated with APSP severity.

## 4. Discussion

To the best of our knowledge, there were few predictive prospective studies on APSP for gastrointestinal surgery. In this study, we assessed possible predictors for APSP after gastrointestinal surgery. We discovered that higher preoperative anxiety and higher expected postsurgical pain score were independently associated with stronger postsurgical pain intensity and severity. Moreover, the study showed that more analgesics were consumed in patients undergoing moderate or severe postoperative pain during the first 24 postoperative hours. Our results confirm and extend those of previous studies focused on patients undergoing other types of surgery.

Despite uniform multimodal analgesic treatment, we discovered a large variability in pain intensity during movement. 59.9% of patients experienced moderate or severe pain during the first 24 postoperative hours, respectively. An important increase in opioid analgesic consumption was observed in the patients experiencing moderate or severe pain compared to those experiencing mild pain. Similar to previous studies on other surgery [9], cumulative analgesic consumption was a strong predictor for APSP. Furthermore, analgesic consumption has significant correlation with patients' factors such as age, depression, anxiety, and expected postsurgical pain.

We found that preoperative anxiety was significantly correlated with APSP intensity and became an independent predictor of APSP intensity and severity. These results are consistent with studies which suggested that presurgical anxiety can predict APSP intensity for hysterectomy [7], cesarean [9], inguinal hernioplasty [10], knee replacement [11], and breast cancer surgery [12–14]. Furthermore, a systematic review in women undergoing breast surgery suggested that presurgical anxiety assessed by HADS, state anxiety, or trait anxiety could predict APSP [15].

Similar to previous studies on cesarean [9, 16] and breast cancer [17, 18], expected postsurgical pain could predict APSP intensity in our study. Meanwhile, it was also a strong predictor of APSP severity. In addition, expected postsurgical pain has been used as predictor of the acute pain trajectories during the first postoperative week after breast cancer surgery. The result suggested that patients with higher expected postsurgical pain intensity had higher pain intercepts (high initial pain intensity) and more unfavorable slopes (poor resolution of pain) [19]. However, a previous trial that explored prediction of APSP following breast cancer surgery showed that expected postsurgical pain was not a risk factor for APSP severity [14]. The reason was probably that the variables included stronger confounding factors such as pain sensitivity, anxiety, and axillary dissection.

Our results did not support an independent association between preoperative depression and APSP, although preoperative depression was correlated with APSP intensity by Pearson's test or univariable analysis. As previously stated, depression was not predictive of APSP severity at 2 days in women with breast cancer surgery, and only body image score emerged as an independent predictor [38]. In addition, a systematic review [15] did not find an association between preoperative depression and APSP in women undergoing breast surgery. But it had an association with CPSP at 3, 6, or 12 months [39, 40]. Thus, the preoperative depression might better predict CPSP compared to APSP.

A significant increase in postsurgical pain intensity was observed with the elevation of SFQ-s and SFQ-l. In addition, SFQ-s and SFQ-l could increase the incidence rate of the clinically meaningful postsurgical pain. However, after adjusting the confounding factors, they both could not independently predict the APSP intensity or severity. Unlike previous study [22], SFQ-s was an important predictor of APSP for day-case surgery, but SFQ-l could not predict APSP. The different outcomes probably came from the different criterion of SFQ, definition of APSP, and type of surgery.

Previous trials showed that preoperative chronic pain was a clinical factor of interest in predicting APSP [7, 10, 22]. However, unlike the previous trials, our study found that there was not relationship between preoperative chronic pain and APSP. The reason may lie in the different way of defining or assessing preoperative chronic pain. Our study used NRS to assess chronic pain. In a previous study, however, it was assessed through brief pain inventory. We defined the preoperative chronic pain as pain in any part of body for 3 months. But in the previous study, the chronic pain was preoperative pain in the surgical area or related to the cause of surgery. Other potential predictors such as female [6], young age [2, 7, 8], type of surgery [17, 18, 21], and duration of surgery [23] were strongly associated with APSP in several studies, respectively. But we could not find their predictive effect for APSP intensity or severity in our study.

According to our study, despite negative correlation with APSP intensity, age was not independently associated with APSP after controlling confounding factors. This may be explained by the uneven distribution of age, because most participants were elderly. In addition, there was no significant difference on APSP in different sites of surgery (stomach, colorectum, and small bowel) and types of surgery (laparotomy and laparoscopy). However, in some studies for breast cancer, type of surgery could predict APSP, because axillary lymph nodes dissection caused potential injuries to the sensory intercostobrachial as compared with other types of surgery.

Our study had several methodological limitations. First, our subjects were patients undergoing gastrointestinal surgery. Whether the results are applicable to other surgeries remains unknown. Second, all psychological predictor variables were assessed at the first day before surgery when the threatening surgical event might influence these variables and their relations to each other. Thus, the results should be considered with caution when compared with studies assessing the risk factors several days or even weeks before surgery. Third, the outcome variable, APSP intensity or severity, was assessed only within the first 24 hours after surgery. We did not frequently assess the pain over several days, so we did not make a clinically meaningful assessment of different pain trajectories among patients. Finally, as we investigated the predictors of APSP only in one single hospital, whether the results can be generalized to other countries should be considered cautiously. As a result, a larger multicenter study is needed.

## 5. Conclusion

In conclusion, this study has shown that cumulative analgesic consumption, preoperative anxiety, and expected postsurgical pain were independently associated with APSP intensity following gastrointestinal surgery. Meanwhile, the predictors were highly associated with APSP severity. Further study is needed to confirm these findings in participants of multicenter clinical trials and different surgical procedures. Future strategies to relieve APSP may start from preoperative psychological interventions.

## Figures and Tables

**Table 1:** Demographic and clinical characteristics of patients.

Characteristics	
Female	110 (39%)
Age, year	65.8 ± 12.0
BMI, kg/m^2^	25.0 ± 5.0
No occupation	225 (79.8%)
Education level	
Primary	99 (35.1%)
Middle or high school	135 (47.9%)
College or above	48 (17.0%)
Marital status	
Single, separated or divorced	38 (13.5%)
Married	244 (86.5%)
Smoking	
Never smoker	157 (55.7%)
Former	58 (20.6%)
Current	67 (23.8%)
Alcohol drinking	
Never drinks	156 (55.3%)
Former	65 (23.0%)
Current	61 (21.6%)
Hypertension	131 (46.5%)
Diabetes	38 (13.5%)
Coronary heart disease	25 (8.9%)
ASA physical status	
ASA I	45 (16.0%)
ASA II	202 (71.6%)
ASA III	35 (12.4%)
Preoperative chronic pain	66 (23.4%)
Previous surgery	143 (50.7%)
Site of surgery	
Stomach	118 (41.8%)
Colorectum	145 (51.4%)
Small bowel	19 (6.7%)
Type of surgery	
Open	199 (70.6%)
Laparoscopic	83 (29.4%)
Duration of surgery, min	180.0 ± 61.8
MME consumption, mg	48.3 ± 16.4
Number of drainage tubes	
1 tube	120 (42.6%)
≥2 tubes	162 (57.4%)
Retention time of tube, day	7.9 ± 3.0
Length of hospital stay, day	18.8 ± 6.8
Malignant tumor	238 (84.4%)

Continuous variables are presented as mean ± standard deviation. Categorical variables are presented as *n* (%). BMI: body mass index; ASA: American Society of Anesthesiologists; MME: morphine milligram equivalent.

**Table 2:** Psychological characteristics of patients.

Characteristics	
HADS: anxiety	3.0 (1.0, 6.0)
HADS: anxiety	
No	226 (80.1%)
Yes	56 (19.9%)
HADS: depression	2.0 (1.0, 5.0)
HADS: depression	
No	243 (86.2%)
Yes	39 (13.8%)
Expected postsurgical pain intensity	5.0 (3.0, 7.0)
Expected postsurgical pain severity	
No or mild	95 (33.7%)
Moderate	110 (39.0%)
Severe	77 (27.3%)
SFQ-s	9.0 (3.0, 15.0)
SFQ-l	6.0 (2.0, 14.0)

Continuous variables are presented as median (interquartile range). Categorical variables are presented as *n* (%). HADS: Hospital Anxiety and Depression Scale; SFQ-s: Surgical Fear Questionnaire short-time consequences; and SFQ-l: Surgical Fear Questionnaire long-time consequences.

**Table 3:** Pearson's correlation between age, BMI, MME consumption, psychological characteristics, and APSP intensity.

Variables	A	B	C	D	E	F	G	H	I
APSP intensity (A)	1.0								
Age, year (B)	−1.71^†^	1.0							
BMI, kg/m^2^ (C)	−0.13	−0.08	1.0						
MME consumption (D)	0.63^†^	−0.14^∗^	0.11	1.0					
HADS: anxiety (E)	0.48^†^	−0.20^†^	0.03	0.58^†^	1.0				
HADS: depression (F)	0.36^†^	0.04	−0.01	0.52^†^	0.53^†^	1.0			
Expected postsurgical pain (G)	0.38^†^	−0.18^†^	0.00	0.56^†^	0.42^†^	0.16^†^	1.0		
SFQ-s (H)	0.31^†^	−0.13^∗^	0.07	0.25^†^	0.57^†^	0.38^†^	0.44^†^	1.0	
SFQ-l (I)	0.31^†^	−0.11	−0.03	0.22^†^	0.55^†^	0.40^†^	0.34^†^	0.75^†^	1.0

^*∗*^*p* < 0.05, ^†^*p* < 0.01. APSP: acute postsurgical pain; BMI: body mass index; MME: morphine milligram milligram equivalent; HADS: Hospital Anxiety and Depression Scale; SFQ-s: Surgical Fear Questionnaire short-time consequences; and SFQ-l: Surgical Fear Questionnaire long-time consequences.

**Table 4:** Univariate and multivariate analyses of predictors for APSP intensity.

Variables	Unadjusted *β* (95% CI)	*p* value	Adjusted *β* (95% CI)	*p* value
Gender				
Male	Reference		0	
Female	0.15 (−0.11–0.42)	0.247	−0.11 (−0.46–0.23)	0.518
Age, year	−0.02 (−0.03 to −0.01)	0.004	−0.01 (−0.03 to −0.01)	0.317
BMI	−0.00 (−0.03–0.02)	0.775	−0.04 (−0.09–0.01)	0.096
Occupation status				
No occupation	Reference		0	0.152
Occupation	0.38 (0.07–0.70)	0.018	0.28 (−0.10–0.66)	
ASA physical status				
ASA I	Reference		0	
ASA II	−0.53 (−0.88 to −0.1)	0.003	0.14 (−0.32–0.6)	0.547
ASA III	−0.42 (−0.89–0.06)	0.088	0.35 (−0.29–0.98)	0.285
Preoperative chronic pain				
Yes	Reference		0	
No	0.29 (−0.01–0.59)	0.059	0.05 (−0.24–0.34)	0.735
Previous surgery				
Yes	Reference		0	
No	−0.17 (−0.43–0.08)	0.179	−0.05 (−0.30–0.20)	0.694
Site of surgery				
Stomach	Reference		0	
Colorectum	−0.17 (−0.44–0.09)	0.204	−0.25 (−0.52–0.02)	0.071
Small bowel	−0.20 (−0.73–0.33)	0.457	−0.25 (−0.79–0.29)	0.365
Type of surgery				
Open	Reference		0	
Laparoscopic	−0.16 (−0.44–0.12)	0.259	0.21 (−0.10–0.52)	0.189
MME consumption, mg	0.07 (0.05–0.09)	<0.001	0.05 (0.03–0.07)	<0.001
HADS: anxiety	0.15 (0.12–0.18)	<0.001	0.12 (0.08–0.15)	<0.001
HADS: depression	0.12 (0.08–0.15)	<0.001	0.11 (−0.04–0.07)	0.565
Expected postsurgical pain	0.20 (0.15–0.26)	<0.001	0.12 (0.06–0.18)	<0.001
SFQ-s	0.04 (0.03–0.05)	<0.001	0.55 (−1.43–2.52)	0.589
SFQ-l	0.04 (0.03–0.06)	<0.001	0.56 (−1.42–2.54)	0.582

BMI: body mass index; ASA: American Society of Anesthesiologists; MME: morphine milligram equivalent; HADS: Hospital Anxiety and Depression Scale; SFQ-s: Surgical Fear Questionnaire short-time consequences; and SFQ-l: Surgical Fear Questionnaire long-time consequences.

**Table 5:** Univariate and multivariate analyses of predictors for APSP severity.

Variables	Unadjusted OR (95% CI)	*p* value	Adjusted OR (95% CI)	*p* value
Gender				
Male	Reference		Reference	
Female	1.44 (0.89–2.35)	0.141	1.31 (0.49–3.48)	0.589
Age, year	0.97 (0.95–0.99)	0.005	0.95 (0.91–1.01)	0.093
BMI	1.0 (0.95–1.05)	0.992	0.91 (0.80–1.03)	0.124
Occupation status				
No occupation	Reference		Reference	
Occupation	1.59 (0.89–2.85)	0.120	1.15 (0.42–3.18)	0.786
ASA physical status				
ASA I	Reference		Reference	
ASA II	0.44 (0.23–0.85)	0.015	1.08 (0.33–3.54)	0.905
ASA III	0.67 (0.28–1.64)	0.383	2.84 (0.49–16.50)	0.244
Preoperative chronic pain				
Yes	Reference		Reference	
No	1.45 (0.83–2.52)	0.193	0.79 (0.37–1.70)	0.541
Previous surgery				
Yes	Reference		Reference	
No	0.73 (0.45–1.18)	0.198	0.95 (0.48–1.87)	0.871
Site of surgery				
Stomach	Reference		Reference	
Colorectum	0.8 (0.49–1.32)	0.385	0.52 (0.26–1.08)	0.079
Small bowel	0.77 (0.28–2.08)	0.602	0.61 (0.12–2.99)	0.539
Type of surgery				
Open	Reference		Reference	
Laparoscopic	0.74 (0.43–1.25)	0.257	1.34 (0.60–3.01)	0.480
MME consumption, mg	1.18 (1.12–1.24)	<0.001	1.15 (1.10–1.21)	<0.001
HADS: anxiety	1.40 (1.28–1.53)	<0.001	1.33 (1.21–1.46)	<0.001
HADS: depression	1.31 (1.20–1.43)	<0.001	1.03 (0.90–1.18)	0.675
Expected postsurgical pain	1.55 (1.36–1.78)	<0.001	1.36 (1.17–1.57)	<0.001
SFQ-s	1.09 (1.06–1.12)	<0.001	1.00 (0.94–1.06)	0.928
SFQ-l	1.09 (1.05–1.12)	<0.001	1.00 (0.95–1.06)	0.922

BMI: body mass index; ASA: American Society of Anesthesiologists; MME: morphine milligram equivalent; HADS: Hospital Anxiety and Depression Scale; SFQ-s: Surgical Fear Questionnaire short-time consequences; and SFQ-l: Surgical Fear Questionnaire long-time consequences.

## Data Availability

The data used to support the findings of this study are available from the corresponding author upon request.
